# Peripheral Arterial Disease in Type 2 Diabetes Is Associated with an Increase in Fibrinogen Levels

**DOI:** 10.1155/2018/3709534

**Published:** 2018-11-08

**Authors:** Qin-Fen Chen, Dan Cao, Ting-Ting Ye, Hui-Hui Deng, Hong Zhu

**Affiliations:** ^1^Medical and Health Care Center, the First Affiliated Hospital of Wenzhou Medical University, Wenzhou 325000, China; ^2^School of the First Clinical Medical Sciences, Wenzhou Medical University, Wenzhou 325000, China; ^3^Department of Endocrinology and Metabolism, the First Affiliated Hospital of Wenzhou Medical University, Wenzhou 325000, China

## Abstract

**Background:**

The present study is undertaken to investigate the fibrinogen levels in type 2 diabetes mellitus (T2DM) and its relation to peripheral artery disease (PAD) based on a more accurate and applied noninvasive measurements of duplex ultrasonography.

**Methods:**

We performed a cross-sectional study including 1096 T2DM patients (474 males and 622 females). The odds ratios (ORs) and 95% confidence intervals (CIs) were presented to show the association between PAD and fibrinogen in the subjects divided by fibrinogen levels quarterly. Furthermore, the univariate and multiple logistic analyses were performed to explore the correlation between PAD and fibrinogen levels, individual components in the cross-sectional study.

**Results:**

Finally, 887 (80.9%) T2DM patients meet the diagnostic criteria of PAD and these patients had considerably higher serum fibrinogen concentration than non-PAD group (*P* < 0.001). Multiple logistic analyses revealed that higher fibrinogen quartiles were positively related with the development of PAD in the adjusted model. After adjusting for known confounding parameters, the ORs for PAD were 1.993 (95% CI: 1.322-3.005, *P* < 0.001), 2.469 (95% CI: 1.591-3.831, *P* < 0.001), and 2.942 (95% CI, 1.838-4.711, *P* < 0.001) for Q2, Q3, and Q4, respectively (all *P* values <0.05).

**Conclusions:**

Our results suggest that serum fibrinogen concentration can be considered as an independent risk factor for PAD in T2DM patients.

## 1. Introduction

Type 2 diabetes mellitus (T2DM) enhances the risk of lower extremity peripheral artery disease (PAD) and hemostatic and inflammatory disturbances with serious consequences on morbidity and mortality [1]. PAD is an expression of systemic atherosclerotic disease [2, 3], and increased evidence has suggested that the inflammatory process plays a major role in initiation, aggravation, and progression of atherosclerosis in diabetic patients [4–7].

Fibrinogen, as one of hemostatic and inflammatory markers, is present in diabetic patients and has generated considerable attention. Previous reports declared that circulating fibrinogen is increased in T2DM [8–11]. Moreover, hyperfibrinogenemia has an established relationship with cardiovascular disease (CVD) in subjects with diabetes mellitus (DM) [12, 13]. Notably, elevated fibrinogen levels are more closely related to PAD in T2DM patients in the majority of reports [14–16], while Hutajulu et al. suggested that fibrinogen level was similar in T2DM patients with PAD and without PAD [17]. Although patients with PAD were determined with an ankle brachial index (ABI) in the previous reports, the medial arterial calcification occurs in some patients suffering from diabetes, which results in an inadequate increase of the assessed ABI and inhibits accurate determination of the ABI. A more accurate and applied noninvasive evaluation for determining PAD in T2DM patients needs to be done.

Based on the relationship of the fibrinogen and PAD which is controversial and the insufficiency of the ABI, more indicators to evaluate whether PAD exists in diabetes are essential. High-resolution duplex ultrasonography provides both anatomic location and functional information about the lower-extremity arterial system and has good sensitivity and specificity as compared with invasive angiography [18–21]. The purpose of this study was to investigate the relationship between serum fibrinogen and PAD in T2DM patients measured by duplex ultrasonography.

## 2. Methods

### 2.1. Study Design

Between June 2008 and July to August 2013, Chinese patients with type 2 diabetes aged 18 years or above who got treatment in the endocrinology department and in line with inclusion criteria at the First Affiliated Hospital of Wenzhou Medical University were selected. Subjects were doctor-diagnosed as T2DM according to 1999 World Health Organization criteria [22]. We performed duplex ultrasonography of the lower limb arteries in enrolled in-patients. Duplex ultrasonography was performed by experienced ultrasonographers using AUSCON Sequoia (Mochida Siemens Medical Systems Co., Tokyo, Japan). In addition, all individuals had obtained the detailed health physical examination, laboratory testing, and an interview on lifestyle, medical history, and medication use with a standard questionnaire. The study protocol was approved by the ethics committee of the First Affiliated Hospital of Wenzhou Medical University.

### 2.2. Physical Assessment

Of all subjects, the clinical research coordinators collected physical assessment data, including age, sex, height, weight, body mass index (BMI), systolic blood pressure (SBP), diastolic blood pressure (DBP), waist circumference, and hip circumference. At every visit, height, weight, waist circumference, and hip circumference were measured by a team of trained nurses in the morning twice during the health examination. While weight was measured without heavy clothing and recorded to the nearest 100 g, height was measured without shoes and recorded to the nearest mm with a stadiometer. Waist circumference and hip circumference were measured to the nearest 1 mm using a stadiometer and a metal anthropometric tape, respectively. BMI was calculated as dividing the weight by the height squared. The waist/hip ratio was calculated as waist circumference divided by hip circumference. Blood pressure was measured at the brachial artery of the person in a seated position after they had had an at least fifteen-minute rest with an automatic instrument (Omron, model 705CP, Kyoto, Japan).

### 2.3. Biochemical Laboratory Test

Blood samples were collected and analyzed from antecubital vein sampling. Among them, laboratory parameters including fibrinogen, high-density lipoprotein-cholesterol (HDL-C), low-density lipoprotein-cholesterol (LDL-C), total cholesterol (TC), triglyceride (TG), and HbA1c were subsequently analyzed by an automated analyzer (Abbott AxSYM, Park, IL).

### 2.4. Definition

In this study, the diagnosis of PAD depended on the duplex ultrasonography examination in reference to Guidelines for the Assessment and Management of Patients with Peripheral Arterial Disease from the American Association in 2005 [23]. According to the intima media thickness, arterial diameter, plaque occurrence, and hemodynamic significance of lesions (femoral artery, superficial femoral artery, popliteal artery, anterior tibial artery, posterior tibial artery, and dorsalis pedis arteries), PAD was defined by qualified, experienced ultrasonic diagnostic experts [24]. The definition of hypertension is SBP ≥ 140 mmHg or DBP ≥ 90 mmHg or previously diagnosed. Moreover, the definition of stroke was diagnosed from professional technician and doctors by computed tomography (CT) [25].

### 2.5. Statistical Analysis

Data analyses were performed with SPSS software (SPSS version 23.0 for Windows) and MedCalc software (MedCalc version 12.7 for Windows). In order to assess the correlation of fibrinogen concentrations and the influence of PAD, the study population was divided into quarters according to the fibrinogen concentration (Q1: ≤3.01 g/L; Q2: 3.02-3.65 g/L; Q3: 3.66-4.55 g/L; and Q4: ≥4.56 g/L). Continuous variables are presented using mean with standard deviation (SD) according to the distribution of data, and categorical parameters were presented as number (%) for incidence rates. Student's *t*-test was used for comparisons of the baseline characteristic in PAD subjects and non-PAD subjects, while 1-way analysis of variance (ANOVA) or the Kruskal-Wallis test for continuous variables and *χ* ^2^ test for categorical variables were used for comparisons in different groups. Odds ratios (ORs) with 95% confidence intervals (CIs) were used to present the relative risks for each category of fibrinogen level with the prevalence of PAD. Multivariable logistic regression analysis was performed to determine whether the association persists after adjusting confounding variables. A two-sided *P* < 0.05 was considered as statistically significant.

## 3. Results

### 3.1. Subject Characteristics

Baseline clinical and biochemical measurements of 1096 subjects (474 males and 622 females) are summarized in Table 1. From the entire study population, 887 (80.9%) cases of PAD were identified. Compared to the non-PAD group, the PAD group has higher mean fibrinogen (4.06 ± 1.39 vs. 3.52 ± 1.20 g/L), lower HDL-C (1.06 ± 0.30 vs. 1.15 ± 0.37 mmol/L), and higher prevalence of hypertension (67.5% vs. 43.5%). In order to analyze the relationship of PAD and fibrinogen, the study population was divided into quarters according to the fibrinogen concentration (Q1: ≤3.01 g/L; Q2: 3.02-3.65 g/L; Q3: 3.66-4.55 g/L; and Q4: ≥4.56 g/L).  Table 2 shows the baseline of the study population according to the quartile measurements of fibrinogen, and in the terms of other parameters, individuals with elevated serum fibrinogen levels lead to a higher prevalence risk of PAD and other details about four quarters of fibrinogen concentration.

### 3.2. Fibrinogen Is Associated with the Prevalence of PAD

All potential variables were initially evaluated in a univariate model to assess the functional relation with the prevalence of PAD. Parameters associated with PAD in the univariate analysis were male, age, smoking, alcohol use, diabetes duration, stroke hypertension, weight, height, BMI, waist circumference, hip circumference, waist/hip ratio, fibrinogen, TC, TG, HDL-C, LDL-C, and HbA1c as shown in Table 3. The selected variables were included in the multivariate model to determine whether these confounding parameters affect the relationship between fibrinogen and PAD. As Table 3 shows, the result of multivariate analysis has presented hypertension (OR = 2.434, 95% CI: 1.774-3.340, *P* < 0.001), waist circumference (OR = 0.982, 95% CI: 0.968-0.997, *P* = 0.016), fibrinogen (OR = 1.332, 95% CI: 1.151-1.540, *P* < 0.001), and HDL-C (OR = 0.510, 95% CI: 0.321-0.811, *P* = 0.004). Finally, fibrinogen was found to be the independent risk factor for the prevalence of PAD in T2DM subjects.

### 3.3. Assessment of the Prevalence of PAD in Subjects Stratified by Different Fibrinogen Levels

To obtain a deeper understanding of the association between different fibrinogen levels and the prevalence risk of PAD in T2DM patients, the ORs for PAD were computed after adjusting for confounding parameters. As Table 4 shows, in model A compared with the subjects in Q1, the ORs for the subjects in Q2, Q3, and Q4 were 2.097 (95% CI: 1.409-3.121, *P* < 0.001), 2.738 (95% CI: 1.799-4.168, *P* < 0.001), and 3.639 (95% CI: 2.317-5.716, *P* < 0.001), respectively. In the adjustment for age and sex (model B), the ORs for PAD were 2.112 (95% CI: 1.419-3.146, *P* < 0.001), 2.765 (95% CI: 1.815-4.213, *P* < 0.001), and 3.651 (95% CI: 2.323-5.738, *P* < 0.001) for Q2, Q3, and Q4, respectively. After adjusting the full confounding parameters (age, sex, hypertension, weight, BMI, waist circumference, hip circumference, TC, HDL-C, and HbA1c), the ORs for PAD were 1.993 (95% CI: 1.322-3.005, *P* < 0.001), 2.469 (95% CI: 1.591-3.831, *P* < 0.001), and 2.942 (95% CI: 1.838-4.711, *P* < 0.001) for Q2, Q3, and Q4, respectively (Table 4). These results suggest that the subjects with elevated fibrinogen levels were associated with higher probability of PAD than subjects with relatively lower hemoglobin concentration in T2DM patients.

### 3.4. Logistic Analysis of the Prevalence of PAD and Fibrinogen in T2DM Patients Stratified by Sex

**Figure 1:**
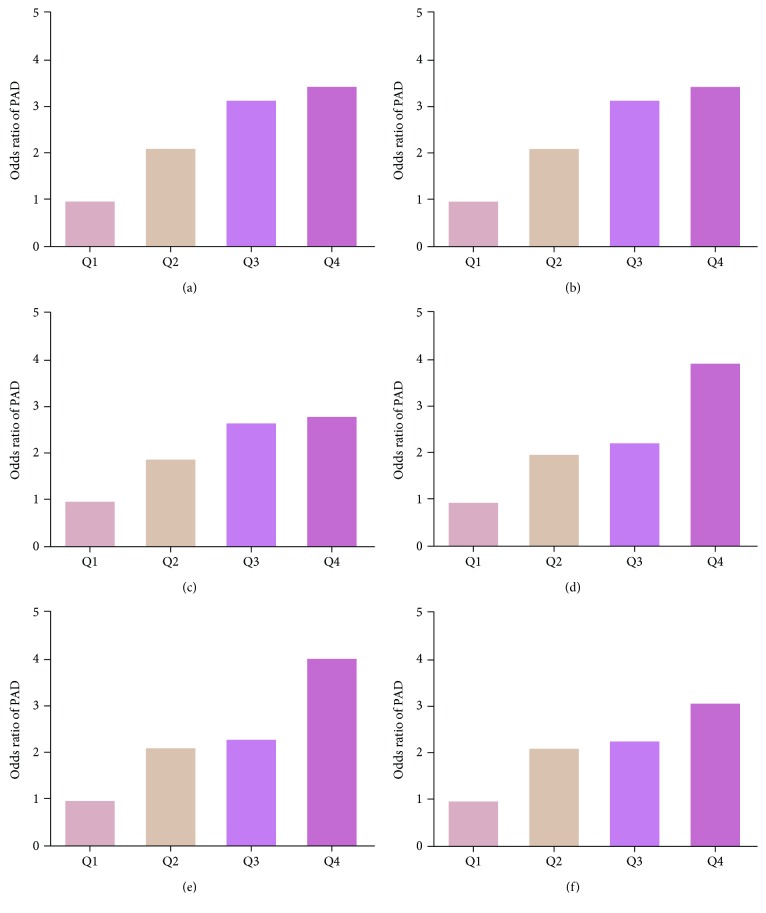
Unadjusted and adjusted odds ratios (ORs) for quartiles of serum fibrinogen in population, stratified by sex: (a-c) male and (d-f) female. Panels (a) and (d) are univariate analysis for fibrinogen; panels (b) and (e) are adjusted for age; panels (c) and (f) are adjusted for age, hypertension, weight, BMI, waist circumference, hip circumference, TC, HDL-C, and HbA1c. All of the *P* values are less than 0.05.

The association between the prevalence of PAD and fibrinogen in 2 diabetes patients was stratified by sex.  Figure 1 shows that individuals with elevated fibrinogen may have higher prevalence of PAD apparently in males and females. As described in Figures 1(c) and 1(f) in the subjects and when compared with the lowest fibrinogen quartile (reference group), males and females with the highest quartile had an OR of 3.119 (95% CI: 1.450-6.707) and 2.869 (95% CI: 1.566-5.256), respectively, after adjusting the full confounding variables.

## 4. Discussion

PAD is a common macrovascular complication in T2DM patients, which not only may contribute for initiation and aggravation of diabetic foot ulcer but also is an efficient predictor of cardiovascular mortality and morbidity [26, 27]. Furthermore, the detection and diagnosis of PAD are difficult as a large proportion of patients remain asymptomatic [28], especially in diabetic patients. It is therefore crucial to gain a greater understanding of potential risk factors of PAD, which may be helpful for early identification and subsequent initiation of therapy for subjects with PAD in T2DM patients.

Our cross-sectional study shows that higher fibrinogen levels are associated independently with PAD based on measurements of duplex ultrasonography in T2DM patients. In our study, after controlling for confounding factors, the ORs for PAD in the Q4 group were nearly three folds compared to individuals of the Q1 group.

Hypercoagulability is present in patients with T2DM, which has been demonstrated to be related to the genesis and development of PAD [29]. Fibrinogen, as a major coagulation protein in the blood, enhances blood and plasma viscosity, platelet activation, and plaque formation [30]. In turn, the effects participate in the pathogenesis of arteriosclerosis. Apart from coagulation processes, fibrinogen may also play a role in inflammatory changes [31]. Fibrinogen is involved in endothelial dysfunction, which is the key pathophysiological mechanism facilitating the process of arteriosclerosis [32–34]. In addition, some studies have implied that fibrinogen values are positively and independently associated with glycemic control, age, hypertension, and components of the metabolic syndrome, which are risk factors for the occurrence of PAD events in T2DM patients [34–36]. Based on these points mentioned above, it is clear that fibrinogen has multiple roles in the pathogenesis of arteriosclerosis.

Methods to evaluate the blood flow in patients with PAD include ABI measurement, vascular ultrasonography, magnetic resonance angiography (MRA), computed tomography angiography (CTA), and angiography. To our knowledge, the diagnosis of PAD was defined by the values of ABI ≤ 0.9 in almost all previous studies. The ABI test is a noninvasive method and is simple to perform [36]. However, it may be falsely negative for calcified, poorly compressible arteries of the lower limb, a very common pathological phenomenon in the elderly and patients with diabetes, which results in artificially elevate ABI values [37]. Duplex ultrasonography imaging is useful for evaluating blood flow of the lower limb arteries and considered as an effective imaging technique for early screening PAD [38, 39]. Compared to MRA, CTA, and angiography, duplex ultrasonography is a safe, inexpensive, widely available, and noninvasive measurement. Moreover, it can provide accurate information about vessels by trained personnel using high-frequency transducers.

Based on these evidences, we performed a cross-sectional study to further assess whether the circulating levels of fibrinogen are associated with subjects with PAD measured by duplex ultrasonography in T2DM patients. The findings of our study reconfirm the positive association between serum fibrinogen levels and PAD. The strengths of this study include the assessment of PAD by a more accurate measurement-duplex ultrasonography and the exhaustive evaluation of biochemical and clinical parameters of atherosclerotic risk. Limitations of this study should also be acknowledged. First, the cross-sectional design limits the ability to establish a cause-effect relationship. Second, a selection bias could have been introduced because of the retrospective nature of the study. In order to minimize this issue, we maximized the sample size included in the study.

## 5. Conclusion

In summary, our observation demonstrated that fibrinogen was positively correlated with the prevalence of PAD in T2DM patients, which should pay more attention to double lower extremity ultrasonic examination in diabetes patients with high fibrinogen level.

## Figures and Tables

**Table 1:** Baseline characteristics between subjects with and without PAD.

	Non-PAD (*N* = 209)	PAD (*N* = 887)	*P* value
Male no. (%)	89 (42.6%)	385 (43.4%)	0.830
Age	62.49 ± 11.70	63.4 ± 11.09	0.294
Smoking (%)	62 (29.7%)	287 (32.4%)	0.453
Alcohol use (%)	54 (25.8%)	197 (22.2%)	0.036
Diabetes duration (months)	115.57 ± 85.03	112.35 ± 81.94	0.612
Stroke (%)	77 (36.8%)	357 (40.2%)	0.362
Hypertension (%)	91 (43.5%)	599 (67.5%)	<0.001
Weight (kg)	65.78 ± 12.19	64.22 ± 11.09	0.073
Height (cm)	162.24 ± 8.57	161.97 ± 8.40	0.676
BMI (kg/m^2^)	24.90 ± 3.64	24.38 ± 3.43	0.053
Waist circumference (cm)	91.88 ± 11.90	90.04 ± 10.41	0.026
Hip circumference (cm)	96.14 ± 8.87	94.87 ± 9.19	0.072
Waist/hip ratio	0.95 ± 0.07	0.96 ± 0.29	0.879
Fibrinogen (g/L)	3.52 ± 1.20	4.06 ± 1.39	<0.001
TC (mmol/L)	4.85 ± 2.55	4.65 ± 1.36	0.115
TG (mmol/L)	1.80 ± 1.39	1.81 ± 1.51	0.928
HDL-C (mmol/L)	1.15 ± 0.37	1.06 ± 0.30	<0.001
LDL-C (mmol/L)	2.71 ± 0.94	2.71 ± 1.02	0.986
HbA1c (%)	9.41 ± 2.24	9.14 ± 2.21	0.114

PAD: peripheral arterial disease; BMI: body mass index; TC: total cholesterol; TG: triglyceride; HDL-C: high-density lipoprotein-cholesterol; LDL-C: low-density lipoprotein-cholesterol.

**Table 2:** Baseline characteristics of 1096 subjects, stratified by quartiles of fibrinogen.

Characteristics	Quartiles of fibrinogen (g/L)				*P* value
	Q1 (≤3.01) *N* = 276	Q2 (3.02-3.65) *N* = 274	Q3 (3.66-4.55) *N* = 274	Q4 (≥4.56) *N* = 272	
PAD no. (%)	188 (68.1%)	224 (81.8%)	234 (85.4%)	241 (88.6%)	<0.001
Male no. (%)	125 (45.3%)	125 (45.6%)	112 (40.9%)	112 (41.2%)	0.533
Age	63.60 ± 10.79	62.81 ± 11.44	62.80 ± 11.17	63.70 ± 11.46	0.668
Smoking (%)	89 (32.2%)	72 (26.3%)	101 (36.9%)	87 (32%)	0.068
Alcohol use (%)	67 (24.3%)	57 (20.8%)	60 (21.9%)	67 (24.6%)	0.661
Diabetes duration (months)	117.64 ± 85.96	107.87 ± 74.67	113.32 ± 87.46	112.98 ± 81.46	0.587
Stroke (%)	105 (38.0%)	110 (40.1%)	107 (39.1%)	112 (41.2%)	0.890
Hypertension (%)	142 (51.4%)	166 (60.6%)	194 (70.8%)	188 (69.1%)	<0.001
Weight (kg)	64.14 ± 11.22	64.63 ± 11.59	65.13 ± 11.36	64.15 ± 11.15	0.700
Height (cm)	161.53 ± 8.42	161.92 ± 8.55	162.5 ± 8.76	162.14 ± 7.98	0.587
BMI (kg/m^2^)	24.49 ± 3.32	24.51 ± 3.73	24.63 ± 3.50	24.29 ± 3.34	0.711
Waist circumference (cm)	90.33 ± 10.53	90.52 ± 10.93	90.63 ± 10.91	90.07 ± 10.61	0.936
Hip circumference (cm)	94.93 ± 8.42	95.06 ± 8.61	95.41 ± 8.58	95.06 ± 10.79	0.938
Waist/hip ratio	0.95 ± 0.07	0.95 ± 0.07	0.95 ± 0.07	0.98 ± 0.51	0.619
TC (mmol/L)	4.67 ± 1.15	4.74 ± 1.34	4.69 ± 1.21	4.65 ± 2.54	0.926
TG (mmol/L)	1.82 ± 1.54	2.03 ± 1.90	1.76 ± 1.11	1.63 ± 1.28	0.021
HDL-C (mmol/L)	1.13 ± 0.32	1.09 ± 0.32	1.10 ± 0.32	0.97 ± 0.29	<0.001
LDL-C (mmol/L)	2.70 ± 0.87	2.70 ± 1.01	2.74 ± 0.94	2.72 ± 1.19	0.975
HbA1c (%)	9.08 ± 2.17	9.14 ± 2.20	9.14 ± 2.27	9.40 ± 2.24	0.340

PAD: peripheral arterial disease; BMI: body mass index; TC: total cholesterol; TG: triglyceride; HDL-C: high-density lipoprotein-cholesterol; LDL-C: low-density lipoprotein-cholesterol.

**Table 3:** Univariate and multivariate analysis of risk factors for PAD.

	Univariate analysis		Multivariate analysis	
	OR (95% CI)	*P* value	OR (95% CI)	*P* value
Sex	1.034 (0.763-1.402)	0.829	1.032 (0.749-1.422)	0.847
Age (year)	1.007 (0.994-1.021)	0.294	1.007 (0.993-1.021)	0.336
Smoking	1.134 (0.817-1.575)	0.453		
Alcohol use	0.820 (0.579-1.160)	0.262		
Diabetes duration (months)	1.000 (0.998-1.001)	0.612		
Stroke	1.155 (0.846-1.577)	0.365		
Hypertension	2.697 (1.983-3.668)	<0.001	2.434 (1.774-3.340)	<0.001
Weight (kg)	0.988 (0.975-1.001)	0.073		
Height (cm)	0.996 (0.979-1.014)	0.676		
BMI (kg/m^2^)	0.959 (0.919-1.001)	0.053		
Waist circumference (cm)	0.984 (0.971-0.998)	0.027	0.982 (0.968-0.997)	0.016
Hip circumference (cm)	0.985 (0.969-1.001)	0.072		
Waist/hip ratio	1.053 (0.542-2.046)	0.879		
Fibrinogen (g/L)	1.443 (1.250-1.665)	<0.001	1.332 (1.151-1.540)	<0.001
TC (mmol/L)	0.939 (0.865-1.020)	0.139		
TG (mmol/L)	1.005 (0.907-1.113)	0.928		
HDL-C (mmol/L)	0.419 (0.270-0.651)	<0.001	0.510 (0.321-0.811)	0.004
LDL-C (mmol/L)	1.001 (0.862-1.163)	0.986		
HbA1c (%)	0.948 (0.887-1.013)	0.114		

PAD: peripheral arterial disease; BMI: body mass index; TC: total cholesterol; TG: triglyceride; HDL-C: high-density lipoprotein-cholesterol; LDL-C: low-density lipoprotein-cholesterol.

**Table 4:** Quartile fibrinogen concentrations and risk of PAD.

	Model A	Model B	Model C
Q1 (≤3.01)	Ref.	Ref.	Ref.
Q2 (3.02–3.65)	2.097 (1.409-3.121)^∗∗^	2.112 (1.419-3.146)^∗∗^	1.993 (1.322-3.005)^∗^
Q3 (3.66–4.55)	2.738 (1.799-4.168)^∗∗^	2.765 (1.815-4.213)^∗∗^	2.469 (1.591-3.831)^∗∗^
Q4 (≥4.56)	3.639 (2.317-5.716)^∗∗^	3.651 (2.323-5.738)^∗∗^	2.942 (1.838-4.711)^∗∗^

Model A is univariate analysis for fibrinogen; model B is adjusted for age and sex; model C is adjusted for age, sex, hypertension, weight, BMI, waist circumference, hip circumference, TC, HDL-C, and HbA1c; ^∗∗^ *P* < 0.001 and ^∗^ *P* < 0.05.

## Data Availability

(1) The clinical data used to support the findings of this study are included within the article. (2) The clinical data used to support the findings of this study are included within the supplementary information file(s). (3) The clinical data used to support the findings of this study are available from the corresponding author upon request.

## Abbreviations

T2DM: Type 2 diabetes mellitus
PAD: Peripheral artery disease
CVD: Cardiovascular disease
DM: Diabetes mellitus
ABI: Ankle brachial index
BMI: Weight, body mass index
SBP: Systolic blood pressure
DBP: Diastolic blood pressure
HDL-C: High-density lipoprotein-cholesterol
LDL-C: Low-density lipoprotein-cholesterol
TC: Total cholesterol
TG: Triglyceride
SD: Standard deviation
ORs: Odds ratios
CIs: Confidence intervals
MRA: Magnetic resonance angiography
CTA: Computed tomography angiography.

## Supplementary Materials

Supplementary Materials Relevant clinical data of the subjects.
